# ELISA for leptospiral 3-hydroxyacyl-CoA dehydrogenase in urine is a promising screening tool for acute leptospirosis

**DOI:** 10.1099/acmi.0.000651.v4

**Published:** 2024-05-31

**Authors:** A.K.U.I. Karunadasa, C. Toma, P. Senaratne, K.G.R.A. Kumara, T.M. Herath, M.M.K. Pathirage, C.D. Gamage

**Affiliations:** 1 Postgraduate Institute of Science, University of Peradeniya, Kandy, Sri Lanka; 2 Department of Bacteriology, Graduate School of Medicine, University of the Ryukyus, Okinawa, Japan; 3 Department of Microbiology, Faculty of Medicine, University of Peradeniya, Kandy, Sri Lanka; 4 Department of Medicine, Faculty of Medicine, University of Peradeniya, Kandy, Sri Lanka

**Keywords:** 3-HADH, ELISA, leptospirosis, urinary biomarker

## Abstract

**Introduction.** Leptospirosis is a zoonotic disease that is prevalent worldwide. Leptospiral 3-hydroxyacyl-CoA dehydrogenase (3-HADH) is excreted in the urine of infected individuals. However, the potential use of 3-HADH as a biomarker for the diagnosis of leptospirosis using enzyme-linked immunosorbent assay (ELISA) has not been investigated. A technique that identifies *Leptospira* in a patient in urine sample will be valuable in regular diagnostics and epidemic scenarios, as opposed to existing serological approaches. This study aimed to develop and evaluate an ELISA that can detect 3-HADH in the urine of patients with confirmed acute leptospirosis and to assess its potential as a screening test for leptospirosis.

**Methods.** Laboratory confirmation of acute leptospirosis was done by *flaB*-nested polymerase chain reaction (PCR) of plasma samples from suspected patients. ELISA-based determination of the presence of 3-HADH in the urine of PCR-positive patients versus PCR-negative patients matched for fever date was performed by coating ELISA plates with urine supernatants and using rabbit anti-3-HADH as the primary antibody. Receiver operating characteristic curve analysis was used to determine the cutoff values for the ELISA. The diagnostic measures between the PCR-positive and PCR-negative patients were compared using the Mann–Whitney U test.

**Results.** In total, 158 febrile patients were assessed, of whom 121 (76.6 %) were male. Of the 15 *flaB-*nested PCR-positive patients, 12 were in the acute phase of the febrile illness. The best cutoff was an average optical density (OD_av_) value of 0.2200 for febrile patients. Sensitivity and specificity were 83.33% [95 % confidence interval (CI), 51.59–97.91 %) and 83.33 % (95 % CI, 76.05–89.13 %), respectively. The OD_av_ values for PCR-positive patients in the acute phase of the disease (≤7 days of fever) were significantly higher than those for PCR-negative patients (*P*<0.001, *U*=114.0, *z*=−4.946).

**Conclusion.** Detection of 3-HADH in urine by ELISA appears to be promising for the screening of acute leptospirosis in suspected patients.

## Data Summary

DDBJ accession numbers of the *Leptospira* sp. *flaB* gene for flagellin, partial CDSs generated from this study are LC752680, LC752681, LC752682, LC752683, LC752684, LC752685, LC752686, LC752687, LC752688, LC752689, LC752690, LC752691, LC752692, LC752693, and LC752694.

## Introduction

Leptospirosis is an emerging zoonotic disease that affects approximately 1.03 million people annually, resulting in 58 900 deaths worldwide [1]. It is caused by a group of spirochetes of the genus *Leptospira* and family *Leptospiraceae* and poses a significant public health concern to both developing and developed countries.

*Leptospira* was previously categorised as saprophytic, intermediate, or pathogenic, correlating with the pathogenicity level. A more thorough and recent phylogenetic study recommended four subclades – P1, P2, S1, and S2 – to replace the previously described clusters of pathogenic (P1), intermediate (P2), and saprophytic (S1 and S2) strains [2]. A clade is a phylogenetic group that includes a common ancestor and all the biological taxa descending from that ancestor. Subclades are separate haplogroup branches identified by the existence of single-nucleotide polymorphisms in comparison to the parent clade.

Pathogenic *Leptospira* colonise the proximal renal tubules. Once they enter the body the incubation period is usually 5–14 days. *Leptospira* are excreted in the urine of host animals and can survive for weeks [3]. Humans are mostly infected with *Leptospira* following skin contact with soil or water contaminated with urine of infected animals [4].

In humans, leptospirosis primarily manifests as an acute febrile illness characterised by fever, chills, headache, myalgia, vomiting, or diarrhoea [5]. Severe manifestations of leptospirosis occur in 5–15 % of human infections and are typified as either Weil’s syndrome or severe pulmonary haemorrhage syndrome, which may present as acute respiratory distress [6].

Clinically suspected cases of leptospirosis are confirmed in the laboratory through a positive blood culture of pathogenic *Leptospira* using dark-field microscopy*,* a positive polymerase chain reaction (PCR) test for pathogenic *Leptospira*, or a microscopic agglutination test (MAT) titre of ≥1 : 320, a fourfold rise, or seroconversion from acute and convalescent sera [7]. Blood culture should be incubated for more than 1 month and up to 3 months before considering a culture as negative due to the slow growth of pathogenic *Leptospira* [8]. Furthermore, identification of pathogenic *Leptospira* in spirochetes-positive cultures requires confirmation by additional tests such as PCR. Thus, culture-based diagnosis of leptospirosis is a time-consuming method that is not applicable to the early diagnosis of this infectious disease. Other molecular methods used for leptospirosis diagnosis, such as quantitative PCR (qPCR), reverse-transcription PCR, and loop-mediated isothermal amplification also require blood samples.

PCR amplification of the nearly full-length virulence flagellin gene (*flaB*) provides an accurate and rapid tool for the identification of leptospires and can be used to rapidly identify animal reservoirs responsible for leptospirosis outbreaks [9]. Thus, it can be applied to clinical diagnosis without leptospiral isolation.

Segawa *et al*. [10] reported that the leptospiral enzyme, 3-hydroxyacyl-CoA dehydrogenase (3-HADH), was detectable in the urine of experimentally infected hamsters prior to the appearance of clinical signs [10]. Based on the findings of Segawa *et al*., leptospiral 3-HADH was detected by Toma *et al*. in urine samples from patients with suspected leptospirosis submitted to the Japanese Reference Laboratories [11]. This enzyme was detected in the urine at the acute phase of infection when leptospiral DNA in the urine was still negative [11]. Given that blood and urine PCR sensitivities are time-dependent, 3-HADH offers an expanded detection window and permits the early diagnosis of leptospirosis when other laboratory tests are negative.

Validation of 3-HADH as a biomarker for leptospirosis diagnosis has not been performed previously in Sri Lanka. Compared to existing serological methods, a diagnostic that detects *Leptospira* in a patient’s urine will be useful in the routine diagnosis and control of disease outbreaks. Therefore, this study aimed to develop and assess the potential of a 3-HADH-based enzyme-linked immunosorbent assay (ELISA) in a Sri Lankan hospital setting with many suspected cases of leptospirosis.

## Methods

### Study population and setting

The study population mainly comprised ‘symptomatic’ patients and ‘asymptomatic’ individuals from the Kandy district of Sri Lanka. The symptomatic population consisted of acute leptospirosis-suspected adult patients (≥18 years of age) admitted to the medical wards of the Teaching Hospital Peradeniya, Sri Lanka. The inclusion criteria for symptomatic individuals were presenting with a complaint of febrile illness (temperature >37.8 °C), headache, myalgia, and physical weakness associated with any of the following symptoms: conjunctival suffusion/conjunctival haemorrhage, meningeal irritation, anuria or oliguria/proteinuria/haematuria, jaundice, haemorrhage, purpuric skin rash, or cardiac arrhythmia/failure. The asymptomatic healthy control group comprised individuals without any complaints of febrile illness.　　

### Sample size

Convenience sampling, a non-probability sampling method in which samples are collected from an easily accessible group from researchers, was applied to select participants from a hospital ward where suspected cases of leptospirosis were admitted. The required sample size was determined using the MedCalc statistical software package (2024 MedCalc Software Ltd). Considering disease prevalence as 10 %, it was planned to recruit 220 clinically leptospirosis-suspected patients for the study.

### Biological sample collection from symptomatic patients

Our laboratory performs *flaB-*nested PCR as a routine test to confirm acute leptospirosis. Symptomatic patients set to undergo *flaB-*nested PCR were asked for their consent to participate in this study by also giving a urine sample in addition to a blood sample. Upon obtaining the patient’s or guardian’s consent, 2 ml of patient blood was collected in EDTA tubes, by trained nurses in the ward. One drop of whole blood was inoculated into 1 ml of liquid Ellinghausen–McCullough–Johnson–Harris (EMJH) medium in a 1.8 ml cryovial and transported at room temperature to the laboratory at the same facility. Clean midstream urine samples were collected in sterile urine bottle. A laboratory request form for PCR test was utilized to gather fundamental clinical and demographic information for the patients. This form, as per our institute’s approval, contained a consent section where patients provided signed informed consent. This consent allowed their clinical and demographic data, as well as the outcomes of their blood and urine samples, to be incorporated into the study.

### Biological sample collection from asymptomatic controls

After obtaining written informed consent, a clean midstream urine sample was collected in a sterile urine container. Blood samples were not collected from the asymptomatic participants as they were non-febrile. The urine samples were transported to the clinical laboratory at the Department of Microbiology, Faculty of Medicine, for processing within 2 h of collection.

### Initial processing and storage of blood samples

EDTA tubes were centrifuged at 3 000 r.p.m. for 5 min. Plasma was separated along with the buffy coat from the EDTA tube and stored at −20 °C in 1.5 ml microcentrifuge tubes.

### Processing and storage of urine samples

All urine samples were transferred into 15 ml tubes in a sterile environment and, then centrifuged at 3 000 r.p.m. for 10 min. Any sediment was discarded, and approximately 10–12 ml of urine supernatant was stored at −20 °C in a fresh 15 ml tube.

### Culturing of *Leptospira* from patient blood samples

Cryovials containing the culture medium and drops of patient blood were stored at room temperature for 1 day. After 24 h, 10 µl of the culture was added to 1 ml of fresh EMJH. These tubes were maintained at 30 °C in a vertical position and examined for the presence of *Leptospira* every 5 days using dark-field microscopy, which was verified by examining their distinctive thin helical structures with prominent hooked ends and motility. After 8 weeks of incubation, samples were classified as negative if no leptospires were detected. If *Leptospira* were found, the cultures were filtered through a 0.2 µm pore size membrane filter, and 0.3 ml of the filtrate was added to fresh tubes containing 3 ml of EMJH media. The same procedure was followed for culturing previously identified species, in which the culture DNA was used as a positive control. Positive cultures were observed and subcultured in EMJH every 2 weeks. Strict sterile conditions were maintained during all culturing steps, which were performed inside a laminar airflow hood.

### DNA extraction from plasma samples

Frozen plasma samples were thawed to room temperature and centrifuged at 13 000 r.p.m. for 30 min. The supernatant was transferred to another 1.5 ml microcentrifuge tube. Genomic DNA was extracted from the pellet using the column-based DNeasy Blood and Tissue kit (QIAGEN, Germany), according to the protocol designated for blood samples. The DNA samples were stored at −20 °C.

### *flaB-*nested PCR to confirm the presence of pathogenic *Leptospira* spp. DNA

Kawabata *et al*. reported that *flaB*-nested PCR can detect pathogenic *Leptospira* strains [12]. In the present study, we used a nested-PCR procedure in order to increase the specificity of detection, as described by Koizumi *et al*. [13]. The amplification was carried out in a thermal cycler (TaKaRa Dice mini, Takara, Japan). The master mix used was FastGene Master Mix (Nippon Genetics, EU) containing *Taq* Polymerase, dNTPs and MgCl_2_. PCR primers l-*flaB*-F1 (5′-CTCACCGTTCTCTAAAGTTCAAC-3′) and l-*flaB*-R1 (5′-TGAATTCGGTTTCATATTTGCC-3′) were used for first-round PCR, and l-*flaB*-F2 (5′-TGTGCACAAGACGATGAAAGC-3′) and l-*flaB*-R2 (5′-AACATTGCCGTACCACTCTG-3′) were used for second-round PCR. These two PCR rounds were performed using 10 µM of each primer and a total reaction volume of 25.0 µl. In the first-round PCR, 2.5 µl of template DNA was used, and the thermal cycle profile was as follows: 95 °C for 25 s followed by 25 cycles at 95 °C for 10 s, 50 °C for 30 s, and 72 °C for 60 s, with a final extension at 72 °C for 5 min. In the second-round PCR, 1.0 µl of first-round PCR product was used as template and the thermal cycle profile was as follows: 95 °C for 25 s followed by 30 cycles at 95 °C for 10 s, 55 °C for 30 s, and 72 °C for 30 s, with a final extension at 72 °C for 5 min. The typical length of target region of the second-round PCR product was 732 bp.

### *flaB* gene sequence determination and phylogenetic analysis

After confirming the amplicons of the *flaB* gene on agarose gels, PCR amplicons were sequenced via the dideoxynucleotide chain termination method using the BigDye Terminator v3.1 Cycle Sequencing kit (Applied Biosystems). GenBank was used to gather the sequences of the 23 additional *Leptospira* species to align and assess the levels of homology. clustalw was used in Molecular Evolutionary Genetics Analysis version 11 (mega 11) [14] to perform multiple sequence alignments of DNA sequences. Phylogenetic distances were calculated using the maximum-likelihood method and the Tamura–Nei model [15].

### Selection of positive and negative samples for the 3-HADH ELISA based on PCR

**Fig. 1.**
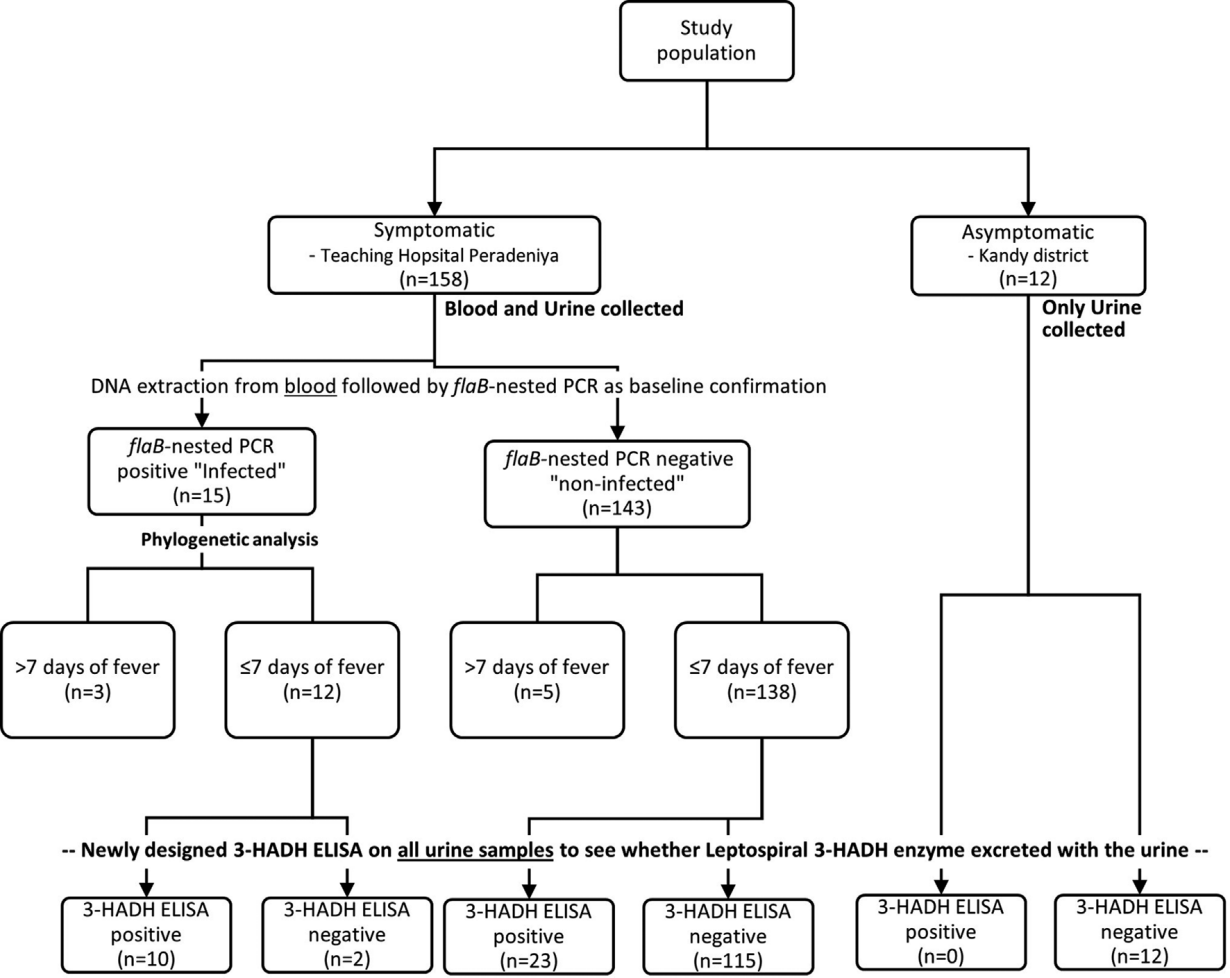
Recruitment of symptomatic and asymptomatic (healthy) participants and selection of urine samples of symptomatic patients to undergo urine 3-HADH ELISA, based on their blood PCR results. 3-HADH, 3-hydroxyacyl-CoA dehydrogenase; ELISA, enzyme-linked immunosorbent assay; PCR, polymerase chain reaction.

The result for the *flaB-*nested PCR was considered as the standard test to confirm the positivity or negativity of each patient sample. A sample was considered to be a true positive when the *flaB-*nested PCR yielded positive results on the agarose gel. The samples then underwent sequencing and phylogenetic analysis with 25 reference strains to determine whether the infecting *Leptospira* species belonged to the P1 or P2 clade. Fig. 1 summarises this categorisation process.

### ELISA for detection of 3-HADH enzyme in urine samples of symptomatic and asymptomatic participants

Urine samples were thawed to room temperature, vortexed for 5 s in 15 ml microcentrifuge tubes, and left upright for 5 min. Then, 0.25 ml of urine was added to each well (2 replicates) of a 96-well microtitre plate (Nunc, Roskilde, Denmark). Further, 1 : 1000 and 1 : 2000 dilutions of purified recombinant 3-HADH enzyme [11] were used as positive controls. Skimmed milk (5 %) (prepared by mixing 5 g of non-fat milk powder in 100 ml distilled water) was used as a negative control. The coated ELISA plate was incubated at 4 °C overnight. The urine samples and controls were discarded, followed by adding 200 µl of blocking buffer (5 % skimmed milk) per well. This was incubated at 37 °C for 1 h. Prior to adding the primary antibody, blocking buffer was discarded, and then the wells were washed twice with 250 µl of Tris-buffered saline Tween 20 (TBST) solution. One gramme of BSA was diluted in 100 ml of 1× phosphate-buffered saline (PBS) solution to prepare the ELISA buffer (EB). Rabbit anti-3-HADH [11] antibody was diluted at a ratio of 1 : 3000 in EB, and 50 µl of the dilution was added in each well of the ELISA plate and incubated at 37 °C. After 1 h, the rabbit anti-3-HADH antibody solution was discarded and then the plate wells were washed thrice with TBST solution. Anti-rabbit IgG horseradish peroxidase (HRP) conjugate was diluted at a ratio of 1 : 5000 in PBS and then 50 µl of the dilution was added to each well and incubated at 37°C for 45 min. The HRP solution was then discarded, and the plate wells were washed with TBST solution six times. O-phenylenediamine dihydrochloride (OPD) solution was prepared by mixing a 10 mg SIGMAFAST OPD tablet (Sigma-Aldrich) with 6 ml distilled water and 4 µl H_2_O_2_. Then, 50 µl of the OPD solution was added into each well. After 5–10 min, following any colour change, 30–50 µl of 1M H_2_SO_4_ solution was added into each well. The wavelength was read at 492 nm.

### Statistical analysis

The patients’ data, PCR results, and ELISA results were recorded using Microsoft Excel 2016. As each urine sample was duplicated during the ELISA, the average optical density (OD_av_) values for the duplicate samples were used for final analysis. Descriptive statistics were used to analyse basic demographics, such as age, sex, and days of febrile illness. Comparisons of the OD_av_ values between the PCR-positive and PCR-negative groups, as well as the healthy group, were performed using an independent samples Mann–Whitney U test. A *P*-value below 0.05 was considered statistically significant at 95 % confidence interval (CI). A receiver operating characteristic (ROC) curve analysis was performed to determine a suitable cutoff value for the newly developed ELISA. These statistical analyses were performed using IBM SPSS version 24.0 software (IBM Corp., Armonk, NY, USA). Finally, diagnostic test parameters were calculated using the MedCalc Diagnostic Test Evaluation Calculator version 22.009, which is freely available online at https://www.medcalc.org/calc/diagnostic_test.php.

### Ethical statement

All participants gave written informed consent to participate in this study. Ethical approval (reference number 2020/EC/20) was obtained from the Ethics Review Committee of the Faculty of Medicine at the University of Peradeniya, Kandy, Sri Lanka.

## Results

### Demographic characteristics of the study participants

A total of 158 febrile patients admitted to the medical wards of the teaching hospital Peradeniya were included in this study. The number of male patients was 121 (76.6 %) and the number of females was 37 (23.5 %). The median age of the cohort was 48.0 years. The majority of the samples (62 %) were collected on the third, fourth, and fifth day of the fever. A total of 12 non-febrile, healthy individuals with no complaints of any illness were included the ‘asymptomatic’ group. Six males (50 %) and six females were recruited. The median age of the asymptomatic cohort was 56.5 years.

### Results of *Leptospira* culture and PCR

A total of 83 blood cultures were observed and none of the patients’ samples used in the ELISA yielded a positive *Leptospira* isolate.

Of the 15 patients whose *flaB-*nested PCR was positive, 12 were in the acute phase of febrile illness, (less than or equal to 7 days of fever). The majority of PCR-positive cases were male (86.67 %). Out of the 143 patients whose *flaB-*nested PCR was negative, 138 were in the acute phase of febrile illness. The band formation at the 732 bp area on the gel was considered a positive PCR result [16].

### Phylogenetic analysis of PCR products

The phylogenetic analysis of sequenced-PCR products showed that the majority (10 samples) belonged to *L. kmetyi* species. Two PCR products belonged to *L. borgpetersenii*, whereas two samples were identified as *L. interrogans*. One PCR product was classified as *L. liceraciae*.

### Serial dilution of purified 3-HADH enzyme

**Fig. 2.**
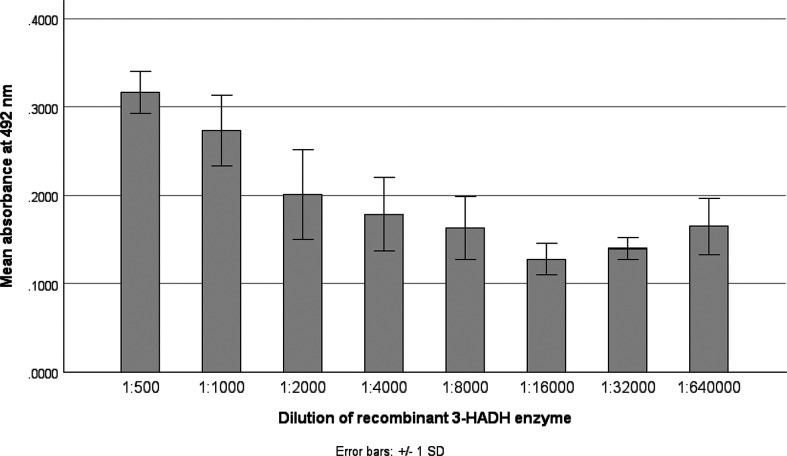
Graph showing decreasing absorbance values at higher dilutions of the of recombinant 3-HADH. 3-HADH, 3-hydroxyacyl-CoA dehydrogenase.

Serial dilutions of the recombinant 3-HADH (515 µg ml^−1^) were used to evaluate the accuracy of the developed ELISA The average absorbance of each sample and its respective duplicate was referred to as OD_av_. Serial dilution beyond 1 : 16 000 showed non-specific binding or background signals. Increasing the dilution decreased the OD_av_ values (Fig. 2).

### ELISA for detecting 3-HADH in urine samples from symptomatic and asymptomatic individuals

The designed 3-HADH ELISA was carried out on urine samples from acute febrile patients and asymptomatic individuals (Fig. 1). The OD_av_ values from the 3-HADH ELISA for the acute febrile-stage patients and asymptomatic individuals were used to calculate the basic statistical indices (Table 1). All febrile patients described here had a fever for ≤7 days.

**Table 1.** Descriptive indices of 3-HADH ELISA results for symptomatic and asymptomatic groups. All symptomatic patients had been febrile for ≤7 days

Statistical indices	Symptomatic		Asymptomatic
	Infected (*flaB-*nested PCR positive) (*n*=12)	Non-infected (*flaB-*nested PCR negative) (*n*=138)	Healthy (*n*=12)
	OD_av_	OD_av_	OD_av_
Mean	0.8934	0.2000	0.1195
Standard deviation	0.6343	0.1960	0.0404
Median	0.7895	0.1513	0.1251
Minimum	0.1664	0.0627	0.0429
Maximum	1.9402	1.6610	0.1836

The mean OD_av_ value for the healthy group was 0.1195, with a standard deviation (sd) of 0.0404. According to the D’Agostino–Pearson test for normal distribution, the OD_av_ values were accepted to be normally distributed (=normality at *P*=0.9694). Therefore, 95 % of the OD_av_ values of the healthy cohort were calculated as the mean+2 sd=0.2003.

**Fig. 3.**
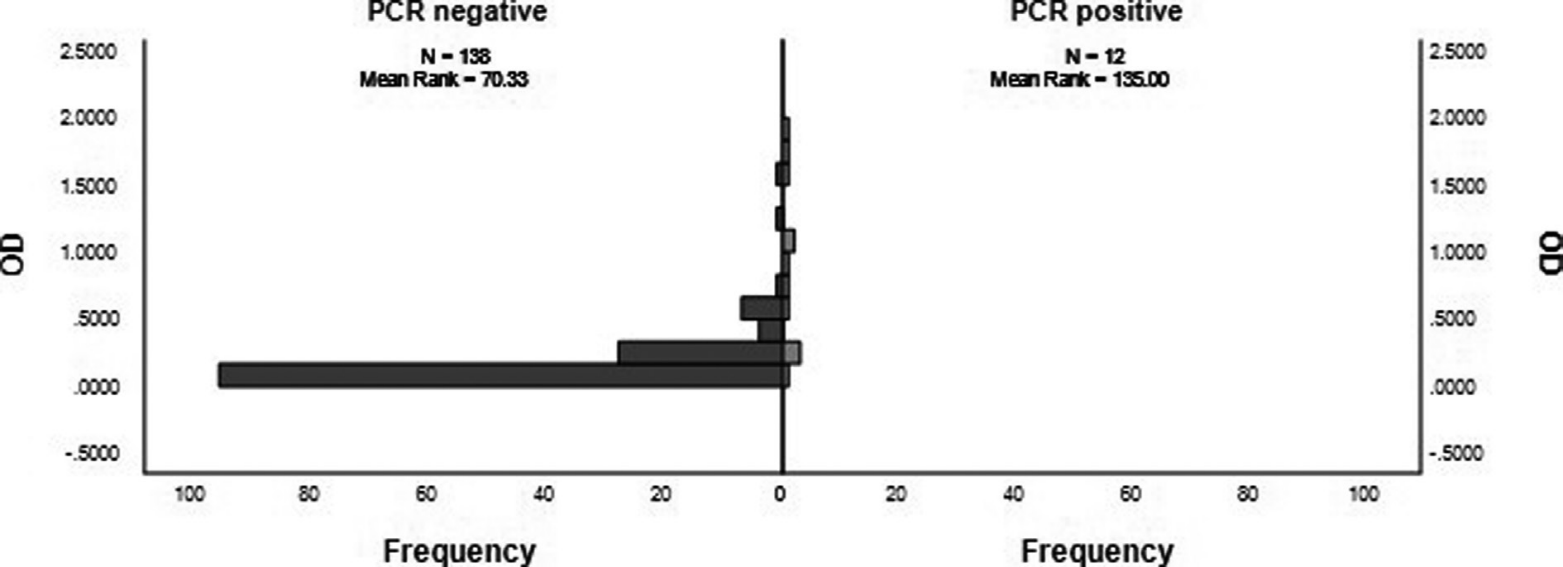
The distribution of OD_av_ values between PCR-positive and PCR-negative samples, as shown by Mann–Whitney U test. All febrile patients described here had a fever for ≤7 days. PCR, polymerase chain reaction; OD_av_, average optical density.

The OD_av_ values for the PCR-positive patients in the acute phase of the disease (median=0.7895, *n*=12) were significantly higher than those for the PCR-negative patients (median=0.1513, *n*=138) (*P*<0.001, *U*=114.00, *z*=−4.946) (Fig. 3).

**Fig. 4.**
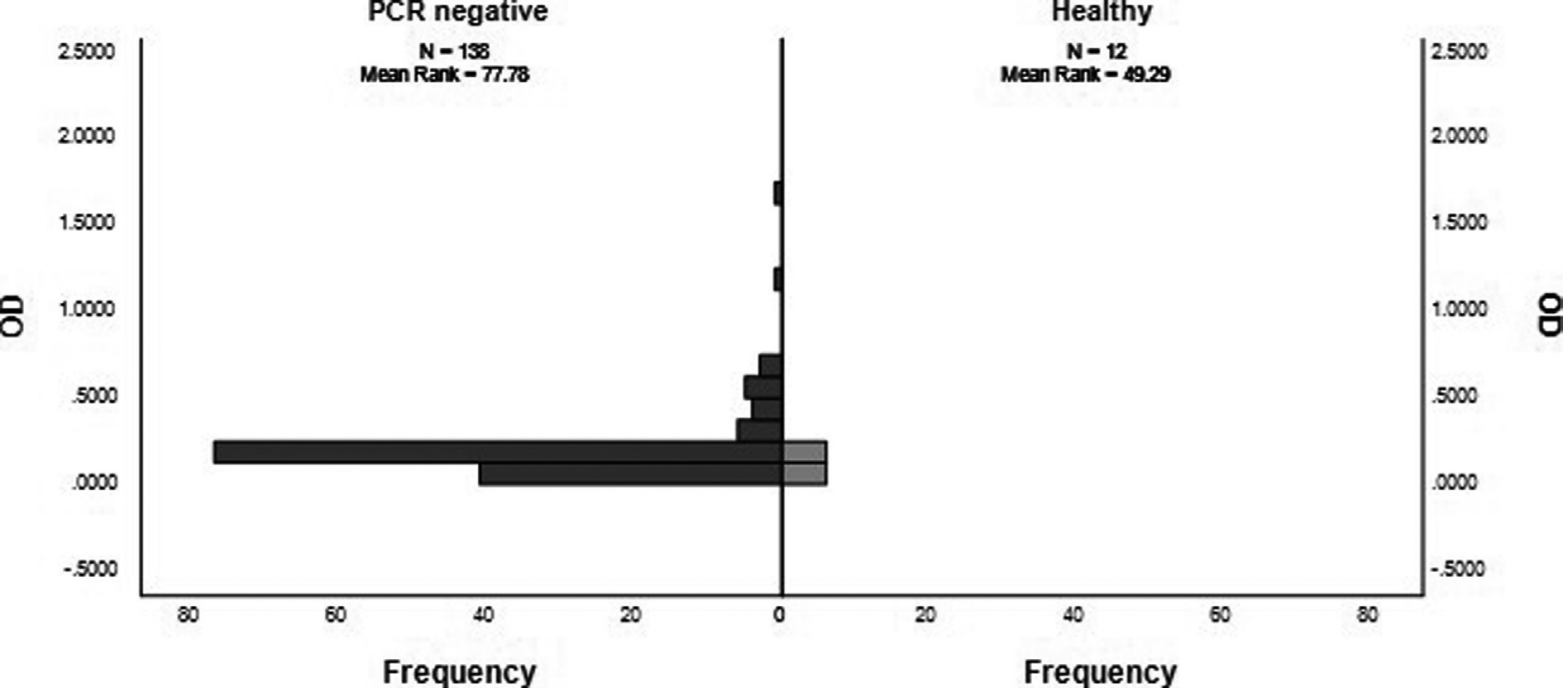
The distribution of OD_av_ values between PCR-negative and healthy groups, as shown by Mann–Whitney U test. All febrile patients described here had a fever day ≤7. PCR, polymerase chain reaction; OD_av_, average optical density.

The OD_av_ values for PCR- negative patients in the acute phase of the disease (median=0.1513, *n*=138), were also significantly higher than those for the healthy group (median=0.1251, *n*=12) (*P*=0.029, *U*=513.50, *z*=−2.178) (Fig. 4).

### ROC curve analysis to obtain cutoff

**Fig. 5.**
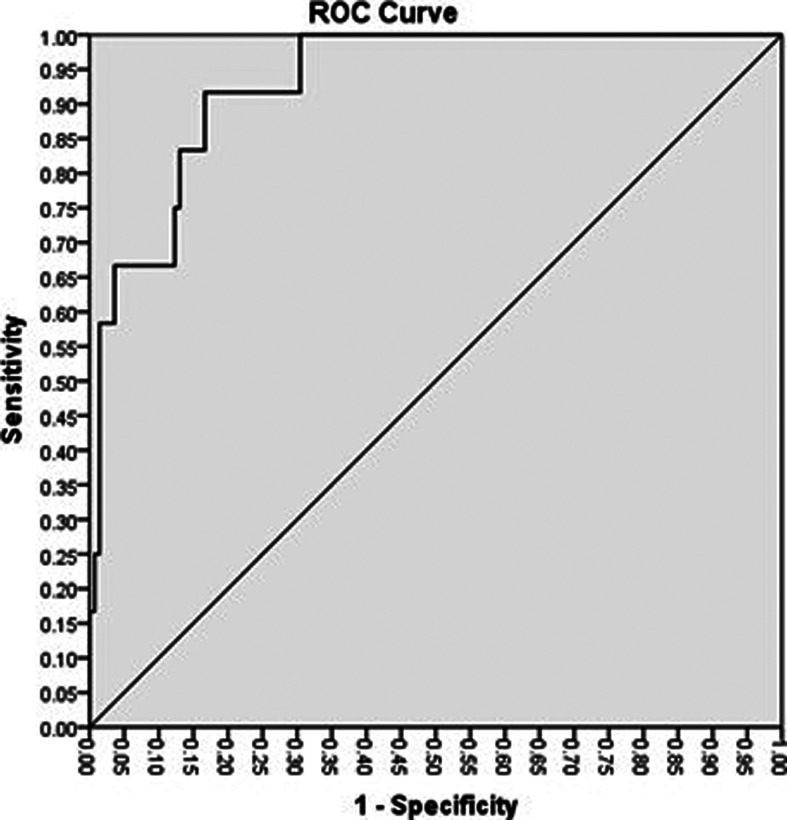
The ROC curve for the OD_av_ values of the 3-HADH ELISA. A total of 138 leptospirosis PCR-negative urine samples and 12 leptospirosis PCR-positive samples were used to construct the ROC curve. The cutoff value is 0.2150 (rounded off to 0.2200) and the area under the ROC curve (AUC) is 0.931(95 % confidence interval=0.874 to 0.988).

The ROC curve analysis (Fig. 5) was conducted using IBM SPSS software version 24.0 (IBM Corp., Armonk, N.Y., USA

The area under the curve (AUC), which indicates accuracy, was determined as 0.931 (95 % confidence interval=0.874–0.988). According to the ROC analysis, the best cutoff was an OD_av_ value of 0.2200. Table 2 shows the positive and negative ELISA results when *flaB-*nested PCR was used as the baseline laboratory confirmation method. The diagnostic parameters based on a cutoff value of 0.2200 and a disease prevalence of 10 % are presented in Table 3.

**Table 2.** Positive and negative ELISA results compared to the standard *flaB-*nested PCR results, with a cutoff OD_av_ value of 0.2200

ELISA result	***Leptospirosis*** as confirmed by *flaB-*nested PCR of symptomatic patients with fever ≤7 days			
	Positive		Negative	
Positive	True positive	10	False positive	23
Negative	False negative	2	True negative	115
Total		12		138

**Table 3.** Diagnostic test parameters for the designed 3-HADH ELISA using from patients with ≤7 days of fever (disease prevalence taken as 10 %)

Statistic	Value	95 %** CI**
Sensitivity	83.33 %	51.59–97.91 %
Specificity	83.33 %	76.05–89.13 %
Positive likelihood ratio	5.00	3.19–7.85
Negative likelihood ratio	0.20	0.06–0.71
Positive predictive value	35.71 %	26.14–46.58 %
Negative predictive value	97.83 %	92.69–99.38 %
Accuracy	83.33 %	76.39–88.91 %

## Discussion

The 3-HADH ELISA method developed in this study showed significantly higher absorbance levels in the urine of patients with confirmed leptospirosis, during the first 7 days of febrile illness. In the preliminary results of the current study presented in 2021, this effect was most pronounced in the first 4 days of febrile illness [17]. Toma *et al.* analysed the presence of leptospiral 3-HADH for the first time in the urine of 70 suspected leptospirosis patients by Western blotting. The enzyme 3-HADH was discovered as early as 2 days after the first onset of the disease and remained detectable for at least 9 days [11]. However, compared to urine PCR and 3-HADH detection in urine, blood PCR was the most sensitive method for diagnosing leptospirosis within 3 days after disease onset [11]. The national guidelines recommend blood PCR for the diagnosis of samples collected within the first 7 days of fever [7]. Thus, our study also used blood PCR as the confirmatory test for acute leptospirosis on samples collected within 7 days of fever. For convenience, we used samples fulfilling the same criteria as for the 3-HADH ELISA.

Out of the 15 patients whose *flaB-*nested PCR was positive, 12 had ≤7 days of febrile illness in the present study. The positivity of these samples was confirmed through phylogenetic analysis. Most patients with suspected and confirmed patients were males, consistent with the current literature. The urine samples of these 12 PCR-positive and 138 PCR-negative patients were analysed using the developed ELISA method to detect the 3-HADH enzyme.

As well-defined positive and negative populations were available for analysis based on the *flaB*-nested PCR, ROC curve analysis was used to obtain the cutoff values for the ELISA results. For the present ELISA method, the cutoff value was set at 0.2200. Although this cutoff value may seem low, it is justified by the fact that the OD_av_ values of PCR-negative samples were significantly lower than those of PCR-positive samples. The antibody binding in ELISA can be improved by increasing the protein concentration. The concentration of fresh urine via filtration can be attempted in future studies.

Of the 158 patients with acute fever in our study, 23 (14.6 %) had false-positive OD values. When there are many false positives and few false negatives, a positive ELISA result is considered a poor indicator of infection, as demonstrated by a positive predictive value of 35.71 % in this study. Moreover, to assess the validity of the 3-HADH ELISA method, urine from a small cohort of asymptomatic individuals was analysed. The mean OD_av_ value of this group was 0.1195, which was below the cutoff value. We also compared of OD_av_ values across PCR-negative and healthy groups and found that the healthy group had a significantly lower value. However, a negative result can be a good indicator of the absence of infection, as shown by the high negative predictive value of 97.83 %. Therefore, the 3-HADH ELISA method developed in the present study can be a useful tool for screening, as it can screen out leptospirosis-negative patients with high certainty.

The purified His-tagged recombinant leptospiral 3-HADH (r3-HADH) enzyme, as described by Toma *et al*. in 2018 [11], was used in the present ELISA method, both in the initial serial dilution and as the positive control in the subsequent ELISA using patients’ samples. The enzyme 3-HADH is a 436-amino acid protein present in both pathogenic and non-pathogenic leptospires. However, an ideal biomarker for the diagnosis of an infectious diseases should be specific to the pathogenic species. Such a problem did not arise in the present ELISA method, because specific antiserum for pathogenic *Leptospira* spp., produced in a previous study by Toma *et al.* [11], was used as the primary antibody (rabbit anti-3-HADH antibody). In *Leptospira*-infected hamsters, an increase in the concentration of different proteins excreted in urine was observed. However, proteins other than leptospiral 3-HADH are not specific to *Leptospira* infection, further demonstrating that detection of leptospiral 3-HADH protein confirms leptospiral infection, but not other diseases.

The reliability of the ELISA method can be influenced by several factors inherent to the technique itself or associated with the patient. First, a cutoff value of 0.22 may help to detect weakly positive urine samples but can also increase the possibility of false-positive results. The baseline determinant of positivity or negativity of the sample – in our case, *flaB-*nested PCR – plays a vital role. In addition, the date of sample collection is crucial in PCR for leptospirosis because bacteraemia reduces after the first week post-infection. Another factor that can affect the 3-HADH ELISA is the administration of antibiotics, which reduces the number of live *Leptospira*. In the present study, three PCR-positive patients had more than 7 days of fever, and the OD values for two of them were lower than the calculated cutoff of 0.2200. One limitation of PCR is that circulating dead *Leptospira* can give a positive result. However, enzyme secretion decreases owing to the reduced number of live bacteria. Although, we collectively calculated the specificity and sensitivity of all samples irrespective of the collection day, classification based on PCR may not be accurate after the seventh day of fever. In fact, the sensitivity of our ELISA increased from 73.33 to 83.33 % when the samples collected after the acute febrile stage were excluded.

A considerable number of patients in the acute febrile phase were PCR-negative. In a tropical country such as Sri Lanka, unexplained febrile illness could mean other common infections, such as hantavirus infection [18], dengue [19], and rickettsia [20]. Because PCR for leptospirosis can only confirm a current *Leptospira* infection, other diseases need to be confirmed with appropriate laboratory tests. In Sri Lanka, routine serological tests for dengue are available but may not be accessible for other infections. Due to the lack of diagnostic facilities in developing countries, the diagnosis of newly recognised or emerging infectious diseases is challenging. In Sri Lanka, there is a need to improve routine diagnostic facilities in hospitals, educate medical professionals, and conduct population-based prospective studies on these diseases.

A curious finding was the detection of *L. kmetyi* in several patient samples. This species is distributed across different environments but has limited evidence of disease causation in humans and animals [21,22]. Although *L. kmetyi* is pathogenic, it is commonly detected in environmental samples [23]. However, based on several reports, the possibility of *L. kmetyi* causing disease cannot be ignored. *L. kmetyi* strain LS 001/16 was isolated from a soil sample associated with a patient with leptospirosis in Kelantan, which is among the states in Malaysia with a high prevalence of leptospirosis [24]. Another report detected *L. kmetyi* DNA in the blood of a patient with leptospirosis who participated in canyoning activities on the Caribbean island of Martinique [25]. A study conducted in the French West Indies detected *L. kmetyi* in blood samples of human patients [26]. Atapattu *et al.* recently detected *L. kmetyi* DNA in urine samples from healthy companion dogs in Sri Lanka [27]. Another notable environmental DNA metabarcoding study found pathogenic *Leptospira* at a higher frequency in Kandy district, with *L. kmetyi* in several samples [28]. Although none of the previous studies in Sri Lanka have detected *L. kmetyi* in human samples, the explained environmental burden could have caused the high number of *L. kmetyi* infections in the present study.

## Limitations and future directions

This study has some limitations. First, attempts to concentrate urine to determine whether the OD values for the 3-HADH signal increased with high protein concentration was futile, as we were unable to import the equipment due to the COVID-19 pandemic. Therefore, concentrated urine should be used in a future study to obtain a higher cutoff value, and this may help reduce the false positives. Increasing the concentration of specific antibodies for 3-HADH is another method that can increase the number of true-positive ELISA results. Notably, rabbit anti-3-HADH antiserum, may contain proteins other than specific antibodies, which may lead to non-specific binding, causing false positives. This problem can be addressed by purifying the antibodies in the antiserum. Such techniques together with qPCR for higher sensitivity can be attempted in a validation study with a larger sample size. Furthermore, the application of 3-HADH ELISA in conjunction with other reported leptospiral antigens could be investigated to develop immunochromatography tests that are useful to healthcare providers, particularly when serological techniques are negative during the acute phase.

In conclusion, the development of this novel ELISA assay targeting the leptospiral enzyme 3-HADH using urine samples presents a promising advancement in the screening for acute leptospirosis. Given that enzyme 3-HADH is exclusively secreted by the leptospiral bacteria, its detection in urine indicates the direct presence of the pathogen, independent of antibody reactions. Implementation of this assay in clinical practice holds the potential to significantly improve routine diagnostic procedures, potentially enabling earlier detection of infection, even prior to the onset of antibody production or during the early stages. This type of detection method is particularly advantageous in situations where drawing blood from a patient is not feasible. This advancement could lead to expedited treatment protocols and improved patient outcomes in clinical settings.

## Abbreviations

AUC: area under the curve
CDS: coding DNA sequence
DALYs: disability adjusted life years
dNTP: deoxyribonucleotide triphosphate
EB: ELISA buffer
ELISA: enzyme-linked immunosorbent assay
EMJH: Ellinghausen-McCullough-Johnson-Harris
3-HADH: 3-hydroxyacyl-CoA dehydrogenase
HRP: horse radish peroxidase
MAT: microscopic agglutination test
ODav: average optical density
PCR: polymerase chain reaction
ROC: receiver-operator characteristic
SD: standard deviation
TBST: tris buffered saline tween

## References

[1] Costa F, Hagan JE, Calcagno J, Kane M, Torgerson P (2015). Global morbidity and mortality of *Leptospirosis*: a systematic review. PLoS Negl Trop Dis.

[2] Vincent AT, Schiettekatte O, Goarant C, Neela VK, Bernet E (2019). Revisiting the taxonomy and evolution of pathogenicity of the genus leptospira through the prism of genomics. PLoS Negl Trop Dis.

[3] Ko AI, Goarant C, Picardeau M (2009). *Leptospira*: the dawn of the molecular genetics era for an emerging zoonotic pathogen. Nat Rev Microbiol.

[4] De Brito T, Silva AM, Abreu PA (2018). Pathology and pathogenesis of human leptospirosis: a commented review. Rev Inst Med Trop Sao Paulo.

[5] Centers for Disease Control and Prevention (2017). About Leptospirosis. https://www.cdc.gov/leptospirosis/symptoms/index.html.

[6] Tubiana S, Mikulski M, Becam J, Lacassin F, Lefèvre P (2013). Risk factors and predictors of severe *Leptospirosis* in New Caledonia. PLoS Negl Trop Dis.

[7] Epidemiology Unit Ministry of Health (2016). National Guidelines on management of Leptospirosis. https://shri.lk/wp-content/uploads/2019/03/lepto_national_guidelines.pdf.

[8] Budihal SV, Perwez K (2014). *Leptospirosis* diagnosis: competancy of various laboratory tests. J Clin Diagn Res.

[9] Wajjwalkul W, Sukmak M, Amavisit P, Sukpuaram T, La-ard A (2015). Molecular characterization of flaB for *Leptospira* identification. Southeast Asian J Trop Med Public Health.

[10] Segawa T, Nomura KH, Villanueva SY, Saito M, Nomura K (2014). Identification of leptospiral 3-hydroxyacyl-CoA dehydrogenase released in the urine of infected hamsters. BMC Microbiol.

[11] Toma C, Koizumi N, Kakita T, Yamaguchi T, Hermawan I (2018). Leptospiral 3-hydroxyacyl-CoA dehydrogenase as an early urinary biomarker of leptospirosis. Heliyon.

[12] Kawabata H, Dancel LA, Villanueva SY, Yanagihara Y, Koizumi N (2001). flaB-polymerase chain reaction (flaB-PCR) and its restriction fragment length polymorphism (RFLP) analysis are an efficient tool for detection and identification of *Leptospira* spp. Microbiol Immunol.

[13] Koizumi N, Muto M, Yamamoto S, Baba Y, Kudo M (2008). Investigation of reservoir animals of *Leptospira* in the northern part of Miyazaki prefecture. Jpn J Infect Dis.

[14] Thompson JD, Gibson TJ, Plewniak F, Jeanmougin F, Higgins DG (1997). The CLUSTAL_X windows interface: flexible strategies for multiple sequence alignment aided by quality analysis tools. Nucleic Acids Res.

[15] Tamura K, Nei M (1993). Estimation of the number of nucleotide substitutions in the control region of mitochondrial DNA in humans and chimpanzees. Mol Biol Evol.

[16] Ogawa H, Koizumi N, Ohnuma A, Mutemwa A, Hang’ombe BM (2015). Molecular epidemiology of pathogenic *Leptospira* spp. in the straw-colored fruit bat (eidolon helvum) migrating to Zambia from the Democratic Republic of Congo. Infect Genet Evol.

[17] Karunadasa AKUI, Toma C, Senaratne K, Kumara K, Gamage CD (2021). Leptospiral 3-hydroxyacyl-CoA dehydrogenase as an early urinary biomarker of *Leptospirosis* in a Sri Lankan setting – interim results. Sri Lankan J Infec Dis.

[18] Dahanayaka NJ, Agampodi SB, Bandaranayaka AK, Priyankara S, Vinetz JM (2014). Hantavirus infection mimicking *Leptospirosis*: how long are we going to rely on clinical suspicion?. J Infect Dev Ctries.

[19] Reller ME, Bodinayake C, Nagahawatte A, Devasiri V, Kodikara-Arachichi W (2012). Unsuspected dengue causes acute febrile illness in rural and semi-urban southern Sri Lanka. Emerg Infect Dis.

[20] Reller ME, Bodinayake C, Nagahawatte A, Devasiri V, Kodikara-Arachichi W (2012). Unsuspected rickettsioses among patients with acute febrile illness, Sri Lanka, 2007. Emerg Infect Dis.

[21] Slack AT, Khairani-Bejo S, Symonds ML, Dohnt MF, Galloway RL (2009). *Leptospira kmetyi* sp. nov., isolated from an environmental source in Malaysia. Int J Syst Evol Microbiol.

[22] Saito M, Miyahara S, Villanueva SYAM, Aramaki N, Ikejiri M (2014). PCR and culture identification of pathogenic *Leptospira* spp*.* from coastal soil in Leyte, Philippines, after a storm surge during Super Typhoon Haiyan (Yolanda). Appl Environ Microbiol.

[23] Ali MR, Safiee AW, Yusof NY, Fauzi MH, Yean Yean C (2018). Isolation of Leptospira kmetyi from residential areas of patients with *Leptospirosis* in Kelantan, Malaysia. J Infect Public Health.

[24] Yusof NY, Muhammad YF, Muhammad HS, Ahmad MN, Khalid MF (2019). Complete genome sequence of *Leptospira* kmetyi sp. LS 001/16, ssolated from a soil sample associated with a *Leptospirosis* patient in Kelantan, Malaysia. Microbiol Resour Announc.

[25] Hochedez P, Escher M, Decoussy H, Pasgrimaud L, Martinez R (2013). Outbreak of *Leptospirosis* among canyoning participants, Martinique, 2011. Euro Surveill.

[26] Bourhy P, Herrmann SC, Theodose R, Olive C, Nicolas M (2013). Serovar diversity of pathogenic *Leptospira* circulating in the French West Indies. PLoS Negl Trop Dis.

[27] Athapattu T, Fernando R, Abayawansha R, Fernando P, Fuward M (2022). Carrier status of *Leptospira* spp. in healthy companion dogs in Sri Lanka. Vector Borne Zoonotic Dis.

[28] Gamage CD, Sato Y, Kimura R, Yamashiro T, Toma C (2020). Understanding leptospirosis eco-epidemiology by environmental DNA metabarcoding of irrigation water from two agro-ecological regions of Sri Lanka. PLoS Negl Trop Dis.

